# Supplementary material for: Peri-Operative Antimicrobial Prophylaxis Modulates CD4+ Lymphocyte Immunophenotype Ex Vivo in High-Risk Patients Undergoing Major Elective Surgery—A Preliminary Observational Study
Source: Antibiotics (Basel). 2025 Oct 14;14(10):1026. doi: 10.3390/antibiotics14101026 (PMC12561684; doi:10.3390/antibiotics14101026)
Supplement: Supplementary file 1 [file antibiotics-14-01026-s001.zip › antibiotics-3872474-supplementary.pdf]

Supplemental Material

Supplemental Methods

Healthy volunteer antibiotic screen

To determine the dose and duration of heat-killed bacteria (E. *coli* (EC)) stimulus required to elicit an immune response in vitro, healthy volunteer (n=16) whole blood was incubated for 6 hours with or without heat-killed E. *coli* (EC). The additional effects of antibiotics were also assessed on EC-stimulated cells using flow cytometry. Granulocytes were gated based on forward- and side-scatter, singlets, live and $CD66b^{+}$, monocytes by HLA-DR positive and then classical monocytes as $CD14^{++}CD16^{-}$. (**Supplemental Figure S11**) Ethical approval was granted by the University College London Research Ethics Committee (REC reference 19181/001).

In response to EC-stimulation, there was an increase in markers associated with activation (CD66b) and intracellular cytokine concentration (IL-1β and IL-6) in granulocytes. Few additional effects were demonstrated with antibiotics; high-dose cefuroxime-metronidazole increased intracellular TNF-α concentration. (**Supplemental Figure S12**)

In response to EC-stimulation, there was an increase in markers associated with chemotaxis (CXCR4 and CCR2), T-cell suppression (PD-L1) antigen presentation (HLA-DR and CD86), and cytokine concentration (IL-1β, IL-6 and TNF-α) in monocytes. When co-incubated with antibiotics; cefuroxime alone and when combined with metronidazole decreased CCR2 expression and high dose cefuroxime reduced HLA-DR expression. (**Supplemental Figure S13**)

Given the lack of antibiotic effect demonstrated at 6 hours in the healthy volunteer model, we performed subsequent time-course experiments in healthy volunteer PBMCs (n=6) stimulated either with EC (monocytes) or CD3/28 beads (lymphocytes) which identified maximal effects on monocyte HLA-DR expression at 24 hours and lymphocyte cell death at 72 hours (at 96 hours there was sharp loss of cell viability). (**Supplemental Figure S14**) These timepoints were chosen for subsequent experiments on patient PBMCs.

Supplemental Tables

Supplemental Table S1: Summary of global antimicrobial prophylaxis use	
**Antimicrobial class**	**Number (%)**
Cephalosporin	160 (47.2)
Penicillin	72 (21.2)
Nitroimidazole	34 (10.0)
Fluoroquinolone	18 (5.3)
Lincosamide	17 (5.0)
Aminoglycoside	10 (2.9)
Macrolide	9 (2.7)
Glycopeptide	8 (2.4)
Nitrofuran	4 (1.2)
Diaminopyrimidines	3 (0.9)
Tetracycline	2 (0.6)
Chloramphenicol	1 (0.3)
Fusidane	1 (0.3)

Data for this table was summarised from the supplemental data of the following meta-analysis on global antimicrobial prophylaxis use: Fowler, A.J., et al., *Liberal or restrictive antimicrobial prophylaxis for surgical site infection: systematic review and meta-analysis of randomised trials.* Br J Anaesth, 2022. **129**(1): p. 104-113)

Supplemental Table S2: Clinical characteristics of whole patient cohort			
**Variable**	Whole cohort (n=83)	Cefuroxime cohort (n=53)	Other antibiotic cohort (n=30)
**Age (years)**	65 (56-72)	65 (55-72)	65 (59-72)
**Biological sex (% male)**	48%	68%	40%
**BMI**	25 (22-29)	25 (22-28)	25 (23-31)
**Co-morbidities**			
Hypertension (%)	33 (40%)	20 (38%)	13 (43%)
Cardiovascular disease (%)	16 (19%)	8 (15%)	8 (27%)
Respiratory disease (%)	24 (29%)	14 (26%)	10 (33%)
Type 2 diabetes (%)	14 (17%)	8 (15%)	6 (20%)
ASA Grade (%)	3 (2-3)	3 (2-3)	3 (2-3)
Active cancer (%)	72 (87%)	47 (89%)	25 (83%)
Cancer staging	2 (2-3)	2 (2-3)	2 (2-3)
Neoadjuvant chemotherapy (%)	43 (52%)	33 (62%)	10 (33%)
**SORT Score (%)**	0.8 (0.4-1.7)	1.1 (0.4-3.2)	0.7 (0.0-1.6)
**Type of surgery**			
Upper GI (%)	48 (59%)	42 (80%)	6 (20%)
Lower GI (%)	12 (14%)	10 (19%)	2 (7%)
Maxillofacial (%)	12 (14%)	0%	12 (40%)
Gynaecological (%)	10 (12%)	1 (2%)	9 (30%)
**Peri-operative antibiotics**			
Prophylaxis administered (%)	83 (100%)	53 (100%)	30 (100%)
Duration of prophylaxis (days)	1 (1-1)	1(1-1)	1 (0-1)
Cefuroxime	2 (2%)	2 (4%)	0%
Cefuroxime and metronidazole	50 (60%)	50 (94%)	0%
Cefuroxime, metronidazole & gentamicin	1 (1%)	1 (2%)	0%
Co-amoxiclav	17 (20%)	0%	17 (57%)
Co-amoxiclav & gentamicin	1 (1%)	0%	1 (3%)
Co-amoxiclav & teicoplanin	1 (1%)	0%	1 (3%)
Co-amoxiclav & metronidazole	1 (1%)	0%	1 (3%)
Ciprofloxacin & clindamycin	2 (2%)	0%	2 (7%)
Ciprofloxacin & metronidazole	5 (6%)	0%	5 (17%)
Ciprofloxacin, metronidazole & teicoplanin	1 (1%)	0%	1 (3%)
Clindamycin	1 (1%)	0%	1 (3%)
Gentamicin	1 (1%)	0%	1 (3%)
**Intra-operative dexamethasone use (%)**	71 (85%)	45 (85%)	26 (87%)
**Operation duration (mins)**	263 (172-349)	255 (163-322)	284 (203-394)
**Blood loss (mls)**	500 (200-500)	500 (200-500)	500 (200-550)
**Peri-operative blood transfusion (%)**	4 (5%)	0%	4 (13%)
**Post-op infection**	37 (45%)	25 (47%)	12 (40%)
Chest	25 (30%)	20 (38%)	5 (17%)
Urine	3 (4%)	3 (6%)	0%
Wound	11 (13%)	5 (9%)	6 (20%)
Other/Unclear	3 (4%)	1 (2%)	2 (7%)
**Clavien-Dindo classification**	2 (1-2)	2 (1-2)	2 (1-2)
**Hospital length of stay (days)**	11 (7.5-17)	11 (8-19)	9 (7-16)
**Death (%)**	4 (5%)	3 (6%)	1 (3%)
Abbreviations: ASA: American association of anaesthesiologists, GI: gastrointestinal			

Supplemental Table S3: Flow cytometry fluorochrome panels

Panel	Associated cell function	Marker	Fluorochrome	Catalogue no	Species	Isotype	[Final]
**Monocytes**	**Gating/ activation**	CD14	BV785	BL 301840	Mouse	IgG2a, κ	1:250
		CD16	BUV395	BD 563785	Mouse	IgG1, κ	1:250
	**Antigen presentation**	HLA-DR	APC-Cy7	BL307618	Mouse	IgG2a, κ	1:250
	**Viability**	LD Aqua	Aqua UV	TF L34957	-	-	1:1000
	**Chemokine receptor**	CCR2 (CD192)	BV711	BL 357232	Mouse	IgG2a, κ	1:250
		CXCR4 (CD184)	BV421	BL 306518	Mouse	IgG2a, κ	1:250
	**Co-stimulation**	CD80	PE	BL 305208	Mouse	IgG1, κ	1:250
		CD86	PE-Dazzle	BL 374218	Mouse	IgG1, κ	1:250
	**T-cell suppression**	PD-L1 (CD274)	APC	BD 563741	Mouse	IgG1, κ	1:250
	**Intracellular cytokine**	IL-1β	FITC	TF 11-7018-42	Mouse	IgG1, κ	1:100
		IL-6	PerCP-Cy5.5	BL 501118	Rat	IgG1, κ	1:100
		IL-10	PE-CY7	BL 501420	Rat	IgG1, κ	1:100
		TNF-α	BUV737	TF 367-7349-42	Mouse	IgG1, κ	1:100
**Lymphocytes**	**Gating/ activation**	CD3	BUV395	BD 564001	Mouse	IgG1, κ	1:250
		CD4	BV785	BL 317442	Mouse	IgG2b, κ	1:250
		CD8	BV711	BD 563677	Mouse	IgG1, κ	1:250
		CD19	APC-Cy7	BD 557791	Mouse	IgG1, κ	1:250
	**Activation**	CD28	BUV737	BD 748475	Mouse	IgG1, κ	1:250
	**Viability**	LD Blue	Blue UV	TF L34962	-	-	1:1000
		PD-1 (CD279)	BV605	BL 329924	Mouse	IgG1, κ	1:250
	**Differentiation**	IL-2	FITC	BL 500307	Rat	IgG2a, κ	1:100
		IL-2R (CD25)	BV421	BD 562442	Mouse	IgG1, κ	1:250
	**Intracellular cytokine**	IFN-γ	BV510	BL 502930	Mouse	IgG1, κ	1:100
		IL-10	PE-CY7	BL 501420	Rat	IgG1, κ	1:100
	**Proliferation**	IL-7R (CD127)	PE	BL 351304	Mouse	IgG1, κ	1:250
	**T-cell suppression**	CTLA-4 (CD152)	PE-Dazzle	BL 349922	Mouse	IgG1, κ	1:250
		PD-L1 (CD274)	APC	BD 563741	Mouse	IgG1, κ	1:250

Abbreviations: APC: Allophycocyanin; BD: Beckton Dickinson; BL: Biolegend; BV: Brilliant Violet; BUV: Brilliant Ultraviolet; CD: Cluster of Differentiation; Cy: Cyanine; FITC: Fluorescein isothiocyanate; HLA-DR: Human leukocyte antigen – DR isotype; LD: Live/Dead; CCR2: C-C motif chemokine receptor 2; CXCR4: CXC motif chemokine receptor 4; IL: Interleukin; IFN: Interferon; PE: Phycoerythrin; PerCP: Peridinin chlorophyll; PD-L1: Programmed death receptor ligand-1; PD-1: Programmed death receptor-1; R: Receptor; TNF: Tissue necrosis factor; TF: Thermo Fisher; UV: Ultraviolet.

Supplemental Table S4: Lymphocyte in-depth flow cytometry fluorochrome panel

Panel	Function	Marker type	Cell Marker	Fluorochrome	Cat no	Species	Isotope	Dilution
**Both**	**Cell gating/ activation**	Cell surface	CD3	SBUV445	BR MCA463	Mouse	IgG1	1:250
		Cell surface	CD4	BUV805	TF 368-0047-42	Mouse	IgG1, κ	1:250
		Cell surface	CD8	APC-Fire 750	BL 301066	Mouse	IgG1, κ	1:250
		Cell surface	CD19	BUV395	TF 363-0198-42	Mouse	IgG1, κ	1:250
**Lymphocyte differentiation**	**Differentiation**	Cell surface	IL-2R (CD25)	SBV570	BR MCA2127	Mouse	IgG1	1:250
		Cell surface	CCR4 (CD194)	BUV563	BD 752566	Mouse	IgG1, κ	1:250
		Cell surface	CCR6 (CD196)	BV786	BD 563704	Mouse	IgG1, κ	1:250
		Intracellular cytokine	IL-2	BV650	BL 500334	Rat	IgG2a, κ	1:100
		Intracellular cytokine	IL-4	PE-Cy7	BD 560672	Mouse	IgG1, κ	1:100
		Intracellular cytokine	IL-17A	APC-R700	BD 565163	Mouse	IgG1, κ	1:100
		Transcription factor	Fox-p3	PE-Cy5	TF 15-4776-42	Rat	IgG2a, κ	1:100
		Intracellular protein	STAT5	RB780	BD 568759	Mouse	IgG1, κ	1:100
		Intracellular protein	T-bet	BV605	BL 644817	Mouse	IgG1, κ	1:100
**T_{h1} function**	**Activation**	Cell surface	CD28	BUV496	BD 741168	Mouse	IgG1, κ	1:250
		Cell surface	HLA-DR	BV711	BD 563696	Mouse	IgG2a, κ	1:250
		Intracellular protein	NF-κB	PE-CF594	BD 565447	Mouse	IgG2b, κ	1:100
	**Viability**	Viability	Live-Dead	Zombie NIR	BL 423106	-	-	1:1000
		Cell surface	Fas (CD95)	BUV615	BD 752346	Mouse	IgG1, κ	1:250
		Cell surface	PD-1 (CD279)	BV480	BD 566112	Mouse	IgG1, κ	1:250
	**Proliferation**	Proliferation	CellTrace	FarRed	TF C34564	-	-	1:1000
		Cell surface	IL-7R (CD127)	PerCP-eFluor 710	TF 46-1278-42	Mouse	IgG1, κ	1:250
	**T-cell suppression**	Cell surface	CTLA-4 (CD152)	AF532	BT NBP2-50286	Mouse	IgG1, κ	1:250
		Cell surface	PD-L1 (CD274)	FITC	BL 393606	Mouse	IgG1, κ	1:250
	**Cytokine concentration**	Intracellular cytokine	IL-10	BUV737	TF 367-7108-42	Rat	IgG1, κ	1:100
		Intracellular cytokine	IFN-γ	BV750	BD 566357	Mouse	IgG1, κ	1:100

Abbreviations: APC: Allophycocyanin, AF: Alexa Fluor, BD: Beckton Dickinson, BL: Biolegend, BR: Biorad, BT: Biotechne, BUV: Brilliant ultraviolet, BV: Brilliant violet, CD: cluster of differentiation, CF: Cyanine-based fluorescent dye, CTLA-4: Cytotoxic T-lymphocyte associated protein-4, Cy: Cyanine, FITC: Fluorescein isothiocyanate, Fox-P3: Forkhead box P3, IFN: Interferon, IL: Interleukin, L/D: Live/Dead, MB: Miltenyi Biotec, NF-κB: Nuclear Factor Kappa B, NIR: Near-infrared, PD-1: Programmed death receptor 1 PD-L1: Programmed death receptor ligand-1, PE: Phycoerythrin, PerCP: Peridinin-chlorophyll-protein, RB: RealBlue, SBV: StarBright violet, SBUV: StarBright ultraviolet, STAT5: Signal transducer and activator of transcription 5, T-bet: T-box transcription factor TBX21, TF: Thermofischer, UV: Ultraviolet.

Supplemental Figures

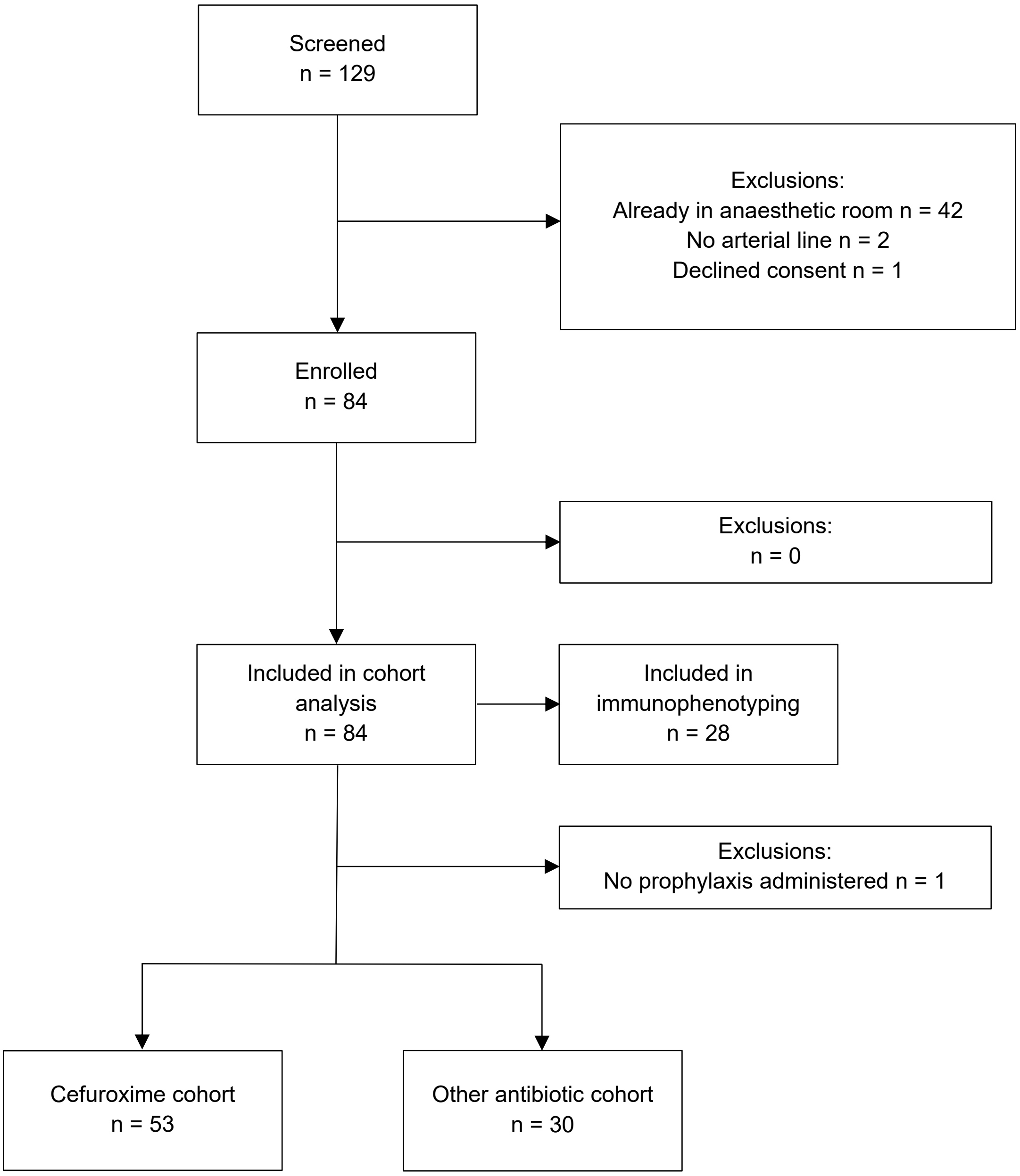

Supplemental Figure S1: Consort diagram of recruited patients

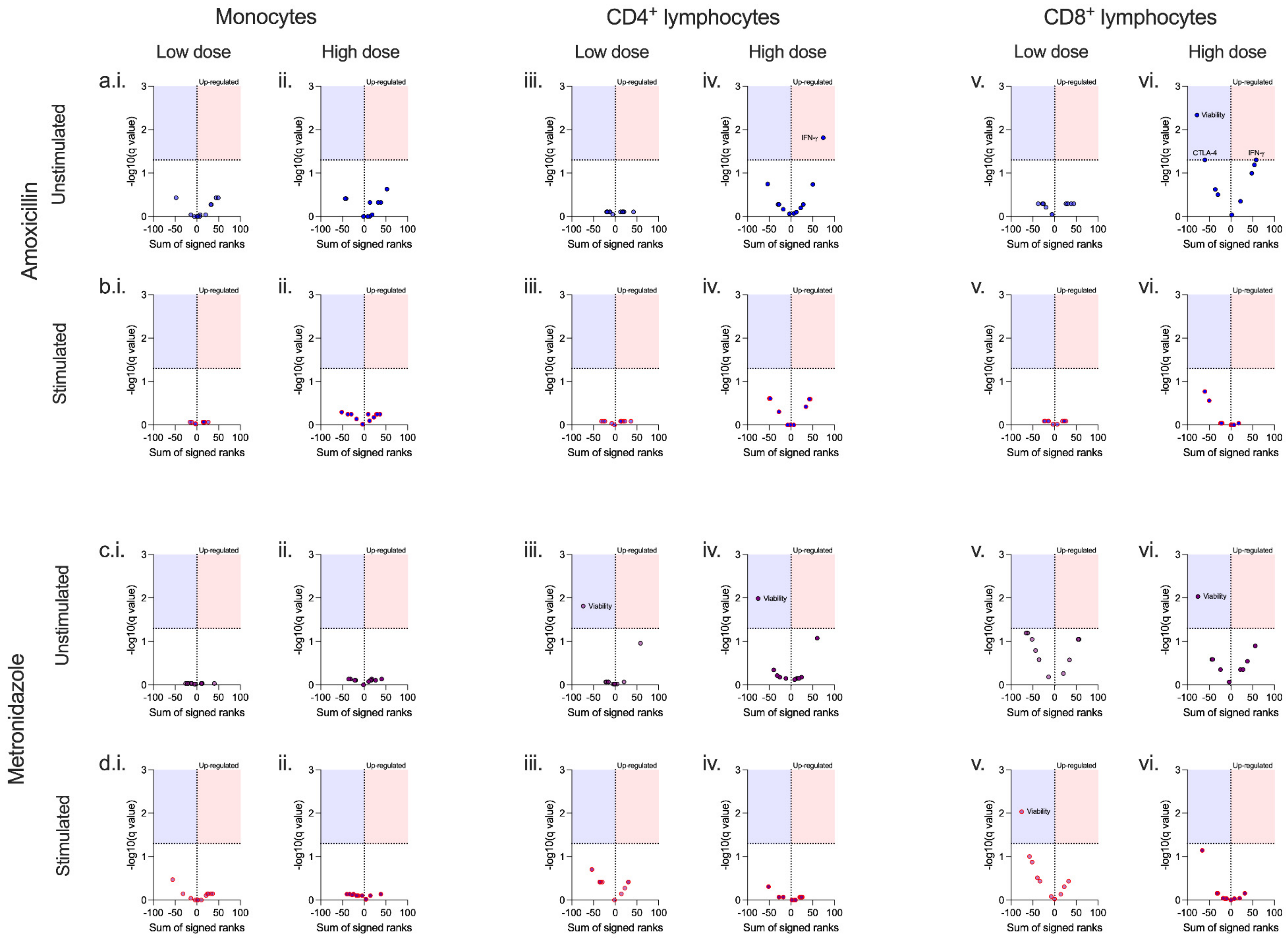

Supplemental Figure S2: Effect of amoxicillin and metronidazole on monocyte and lymphocyte immunophenotype

PBMCs isolated from patients immediately postoperatively (n=12) were incubated with low (5µg/ml, i., iii., and v.) or high (25µg/ml, ii., iv., and vi.) doses of amoxicillin (blue, a. and b.) or metronidazole (purple, c. and d.) alone (black dot border, a. and c.) or with (red dot border, b. and d.) heat-killed E coli (monocytes, 24 hours) or CD3-28 beads (lymphocytes, 72 hours) and the effect on classical monocyte (i. and ii.), $CD4^+$ (iii. and iv.) $CD8^+$ (v. and vi.) lymphocyte immunophenotype assessed. Data expressed as volcano plots generated by calculating a corrected q-value (-log10) using a False Discovery Rate (FDR) of 5% using the two-stage step-up method of Benjamini, Krieger and Yekutieli. Red box represents markers upregulated by antibiotics compared to control or stimulus only, blue box those markers downregulated.

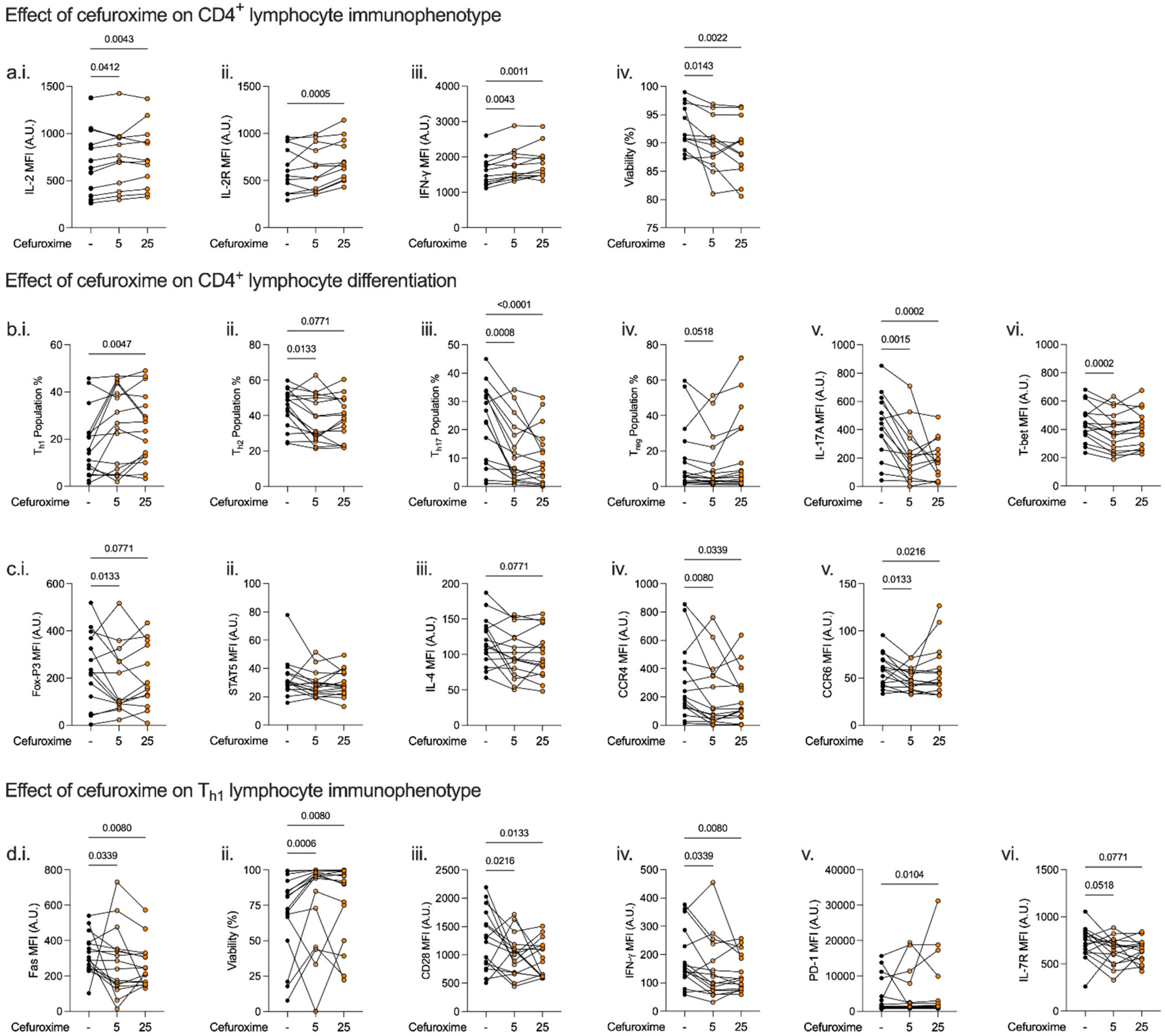

Supplemental Figure S3: Summary of effects of cefuroxime on CD4+ lymphocyte immunophenotype

PBMCs isolated from patients immediately postoperatively (a. n=12, b.-d. n=16) were incubated with low (5µg/ml, light orange) or high (25µg/ml, dark orange) doses of cefuroxime for 72 hours and the effect on CD4+ lymphocyte immunophenotype compared to control (black dot) assessed. Data expressed as slope plots and analysed using Freidman with uncorrected Dunn's test. Only values with p<0.05 shown.

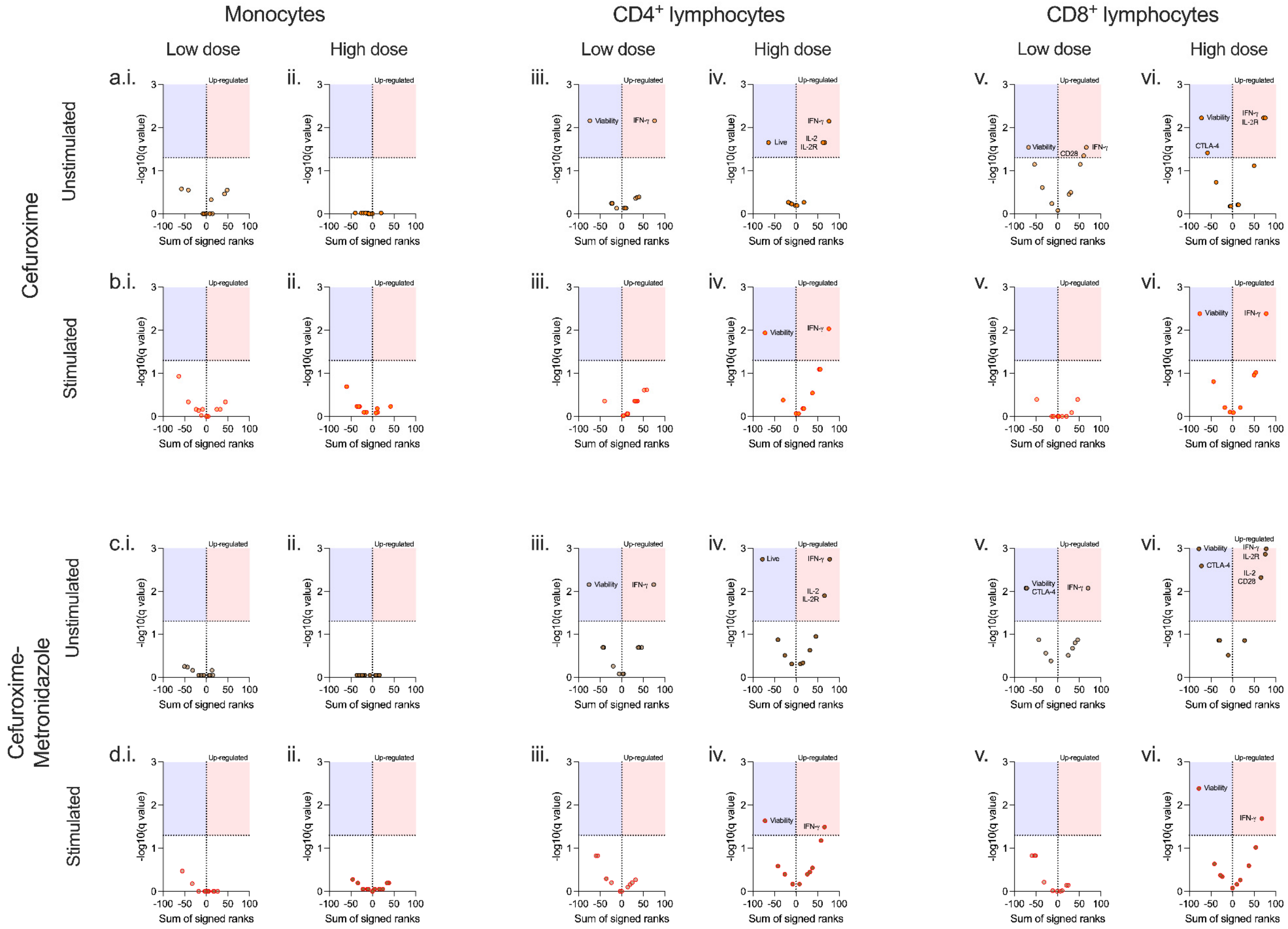

Supplemental Figure S4: Effect of cefuroxime and combined cefuroxime-metronidazole on monocyte and lymphocyte immunophenotype

PBMCs isolated from patients immediately postoperatively (n=12) were incubated with low (5µg/ml, i., iii., and v.) or high (25µg/ml, ii., iv., and vi.) doses of cefuroxime (orange, a. and b.) or combined-cefuroxime-metronidazole (brown, c. and d.) alone (black dot border, a. and c.) or with (red dot border, b. and d.) heat-killed E coli (monocytes, 24 hours) or CD3-28 beads (lymphocytes, 72 hours) and the effect on classical monocyte (i. and ii.), CD4+ (iii. and iv.) CD8+ (v. and vi.) lymphocyte immunophenotype assessed. Data expressed as volcano plots generated by calculating a corrected q-value (-log10) using a False Discovery Rate (FDR) of 5% using the two-stage step-up method of Benjamini, Krieger and Yekutieli. Red box represents markers upregulated by antibiotics compared to control or stimulus only, blue box those markers downregulated

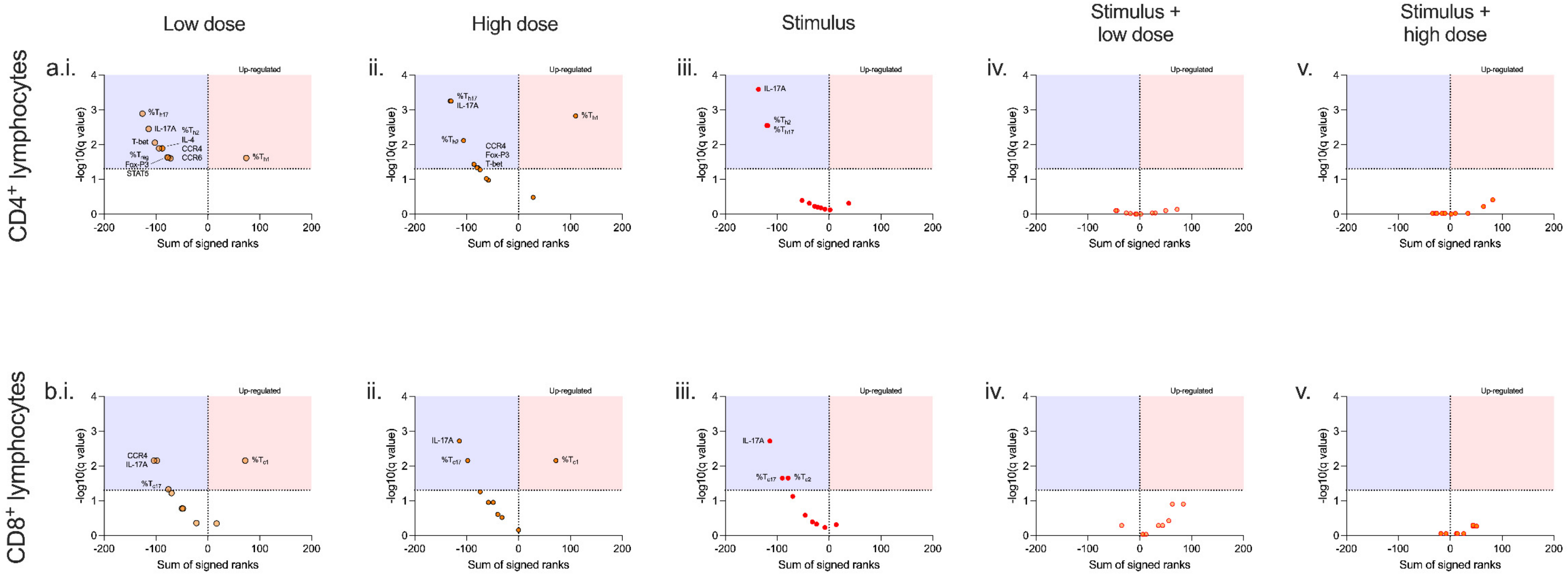

Supplemental Figure S5: Cefuroxime has immunomodulatory effects on lymphocyte differentiation

PBMCs isolated from patients immediately postoperatively (n=16) were incubated with low (5µg/ml, i. and iv.) or high (25µg/ml, ii. and v.) doses of cefuroxime (orange, i., ii., iv, and v.) alone (black dot border, i. and ii.) or with (red dot border, iv. and v.) CD3-28 beads (red dot and border, iii.) for 72 hours and the effect on $CD4^+$ (a.) and $CD8^+$ (b.) lymphocyte subtypes and markers of differentiation assessed. Data expressed as volcano plots generated by calculating a corrected q-value (-log10) using a False Discovery Rate (FDR) of 5% using the two-stage step-up method of Benjamini, Krieger and Yekutieli. Red box represents markers upregulated by antibiotics compared to control or stimulus only, blue box those markers downregulated.

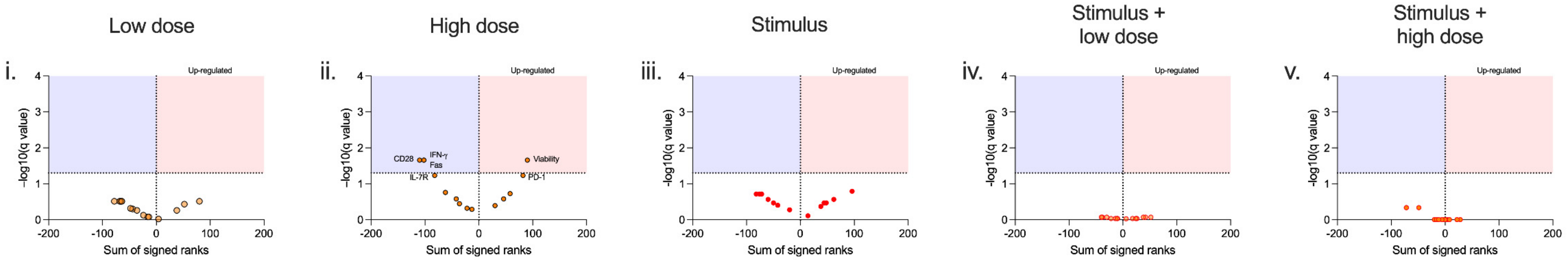

Supplemental Figure S6: Cefuroxime has immunomodulatory effects on T_{h1} lymphocyte immunophenotype

PBMCs isolated from patients immediately postoperatively (n=16) were incubated with low (5µg/ml, i. and iv.) or high (25µg/ml, ii. and v.) doses of cefuroxime (orange, i., ii., iv, and v.) alone (black dot border, i. and ii.) or with (red dot border, iv. and v.) CD3-28 beads (red dot and border, iii.) for 72 hours and the effect on T_{h1} subsets assessed. Data expressed as volcano plots generated by calculating a corrected q-value (-log10) using a False Discovery Rate (FDR) of 5% using the two-stage step-up method of Benjamini, Krieger and Yekutieli. Red box represents markers upregulated by antibiotics compared to control or stimulus only, blue box those markers downregulated.

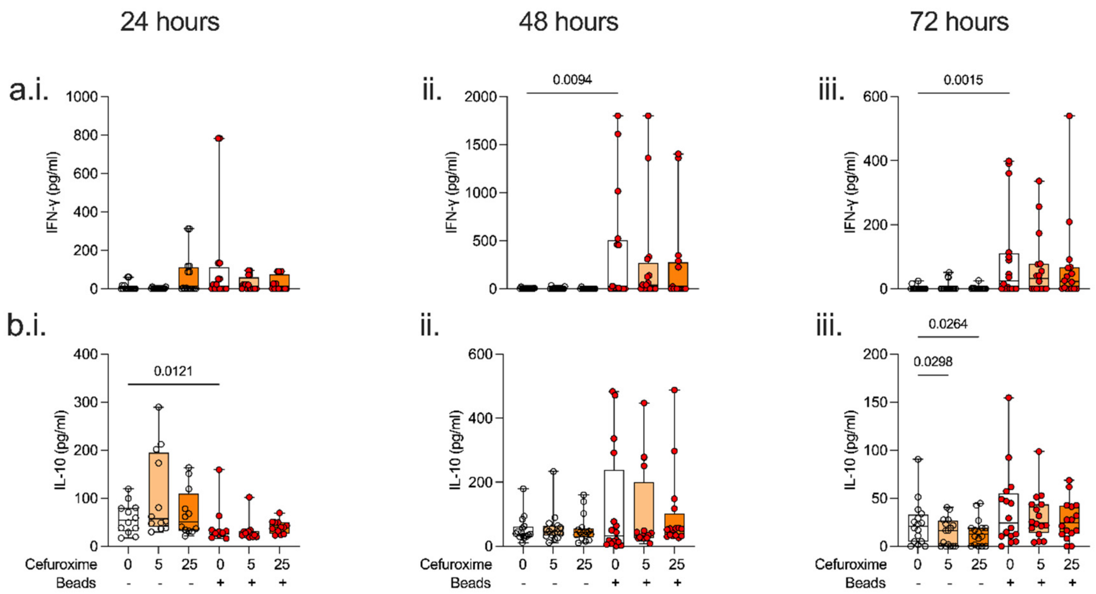

Supplemental Figure S7: Cefuroxime has an immunomodulatory effect on cytokine release

PBMCs isolated from patients immediately postoperatively (n=16) were incubated with low (5µg/ml, light orange) or high (25µg/ml, dark orange) doses of cefuroxime alone (black dot border) or with CD3-28 beads (red dot) for 72 hours and the effect on released IFN-γ (a.) and IL-10 (b.) measured in the supernatant every 24 hours using ELISA. Data expressed as individual replicate (dot), median (horizontal line), interquartile range (box) and range (whisker) and analysed using Friedmans test. Only $p<0.05$ shown.

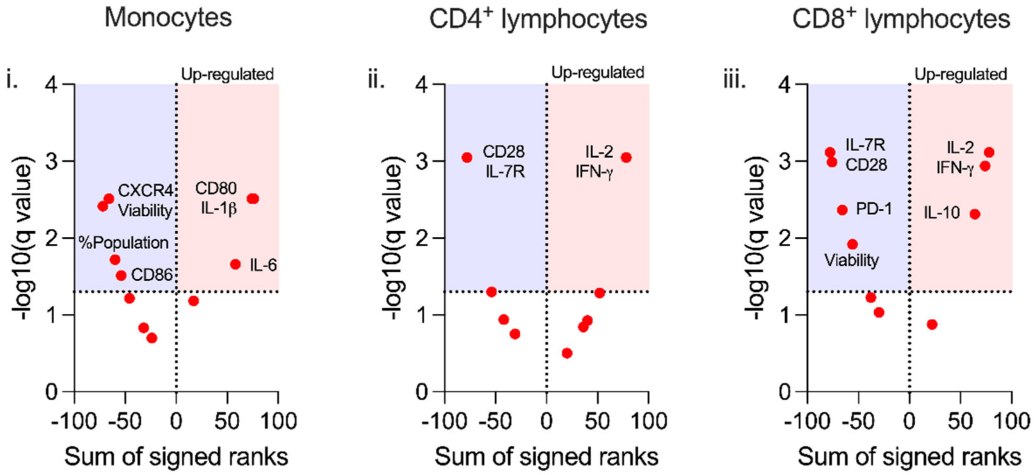

Supplemental Figure S8: Effect of stimulus on monocyte and lymphocyte immunophenotype

PBMCs isolated from patients immediately postoperatively (n=12) were incubated with heat-killed E coli (monocytes, 24 hours) or CD3-28 beads (lymphocytes, 72 hours) and the effect on classical monocyte (a.), $CD4^{+}$ (b.) $CD8^{+}$ (c.) lymphocyte immunophenotype assessed. Data expressed as volcano plots generated by calculating a corrected q-value (-log10) using a False Discovery Rate (FDR) of 5% using the two-stage step-up method of Benjamini, Krieger and Yekutieli. Red box represents markers upregulated by stimulus compared to control, blue box those markers downregulated.

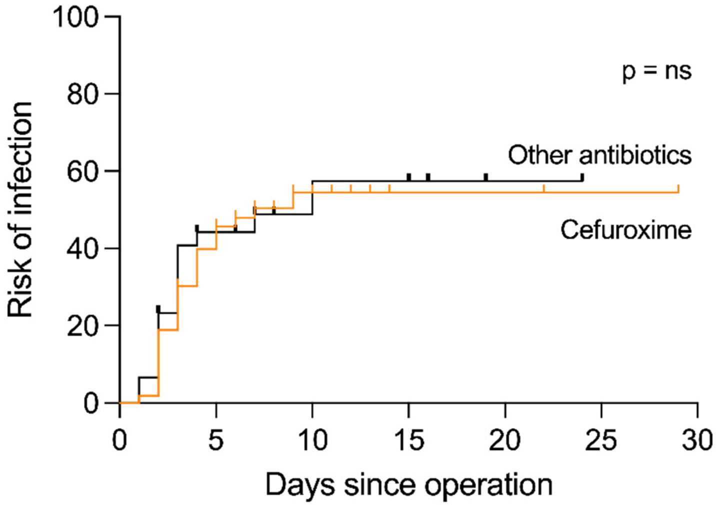

Supplemental Figure S9: The immunomodulatory effects of cefuroxime may not be clinically relevant

The patient cohort was divided into those who had received cefuroxime prior to surgical incision and those who had received other antibiotics and the risk of development of infection (as adjudicated by StEP-COMPAC criteria compared using log-rank test

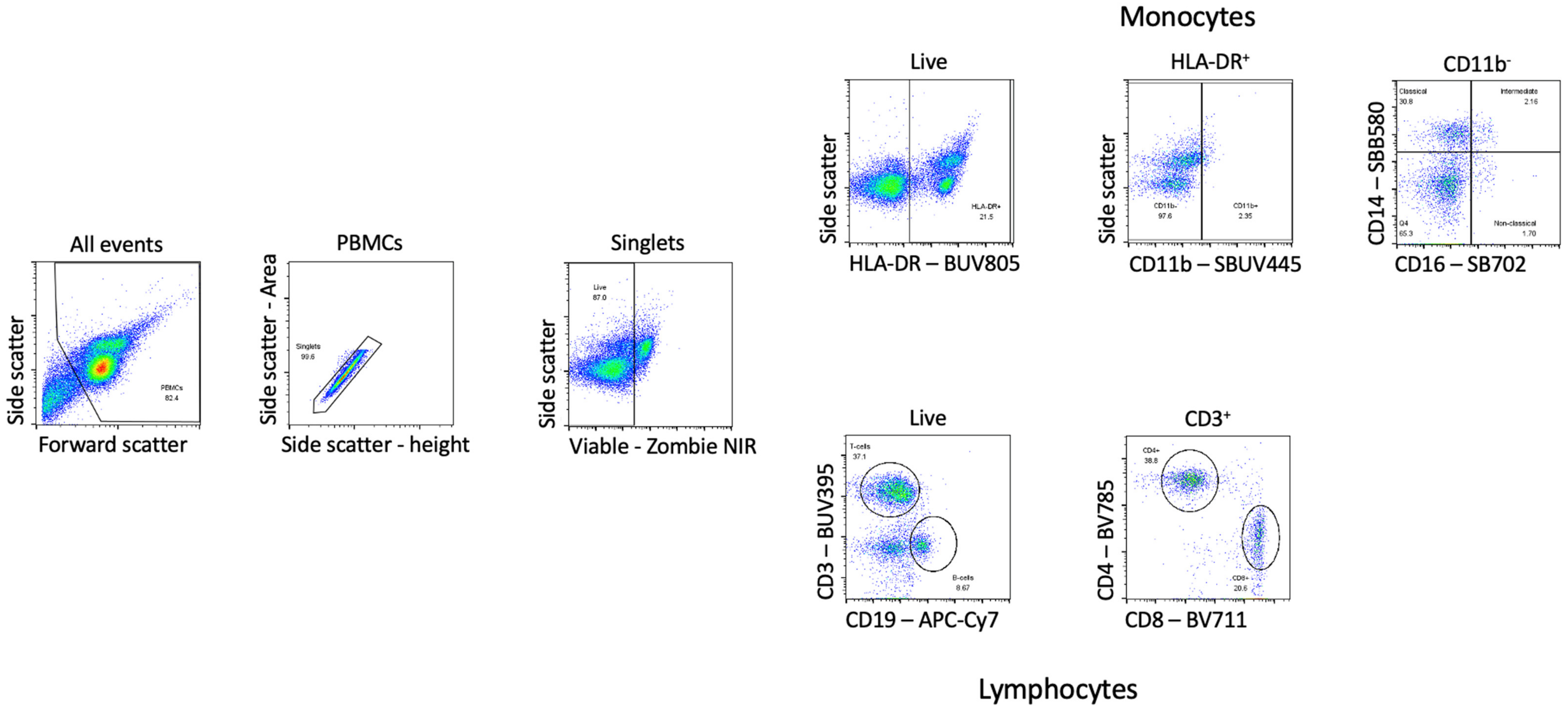

Supplemental Figure S10: Example gating strategy to identify classical monocytes and CD4⁺ and CD8⁺ lymphocytes

PBMCs were identified initially using forward and side scatter, singlets then identified using side scatter area by height, and then viable cells using Zombie near infrared (NIR). Monocytes (top) were then identified as HLA-DR^{+} (Brilliant UV (BUV) 805), CD11b^{-} (StarBright Ultraviolet (SBUV) 402), and finally CD14^{++}/CD16^{-} (StarBright Blue (SBB) 580 and SuperBright (SB) 702 respectively). Lymphocytes (bottom) were differentiated using CD3^{+} or CD19^{+} (BUV395 and Allophycocyanin-Cyanine7 (APC-Cy7), then CD4^{+} or CD8^{+} (Brilliant violet (BV) 785 and BV711 respectively).

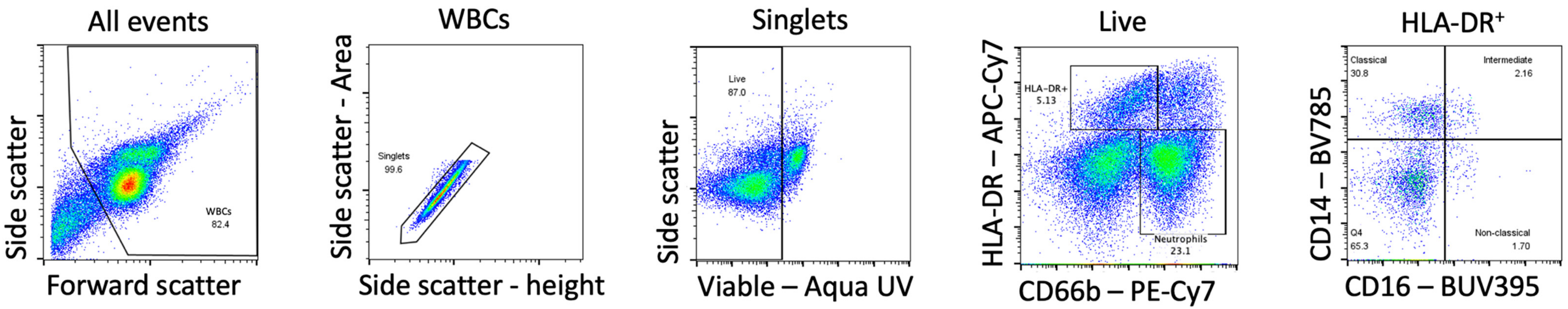

Supplemental Figure S11: Example gating strategy to identify granulocytes and classical monocytes

White blood cells (WBCs) were identified initially using forward and side scatter, singlets then identified using side scatter area by height, and then viable cells using Aqua UV live/dead stain. Granulocytes were then identified as CD66b^{+} (PE-Cy7) whilst monocytes were then identified as HLA-DR^{+} (APC-Cy7), and CD14^{++}/CD16^{-} (BV785 and BUV395 respectively).

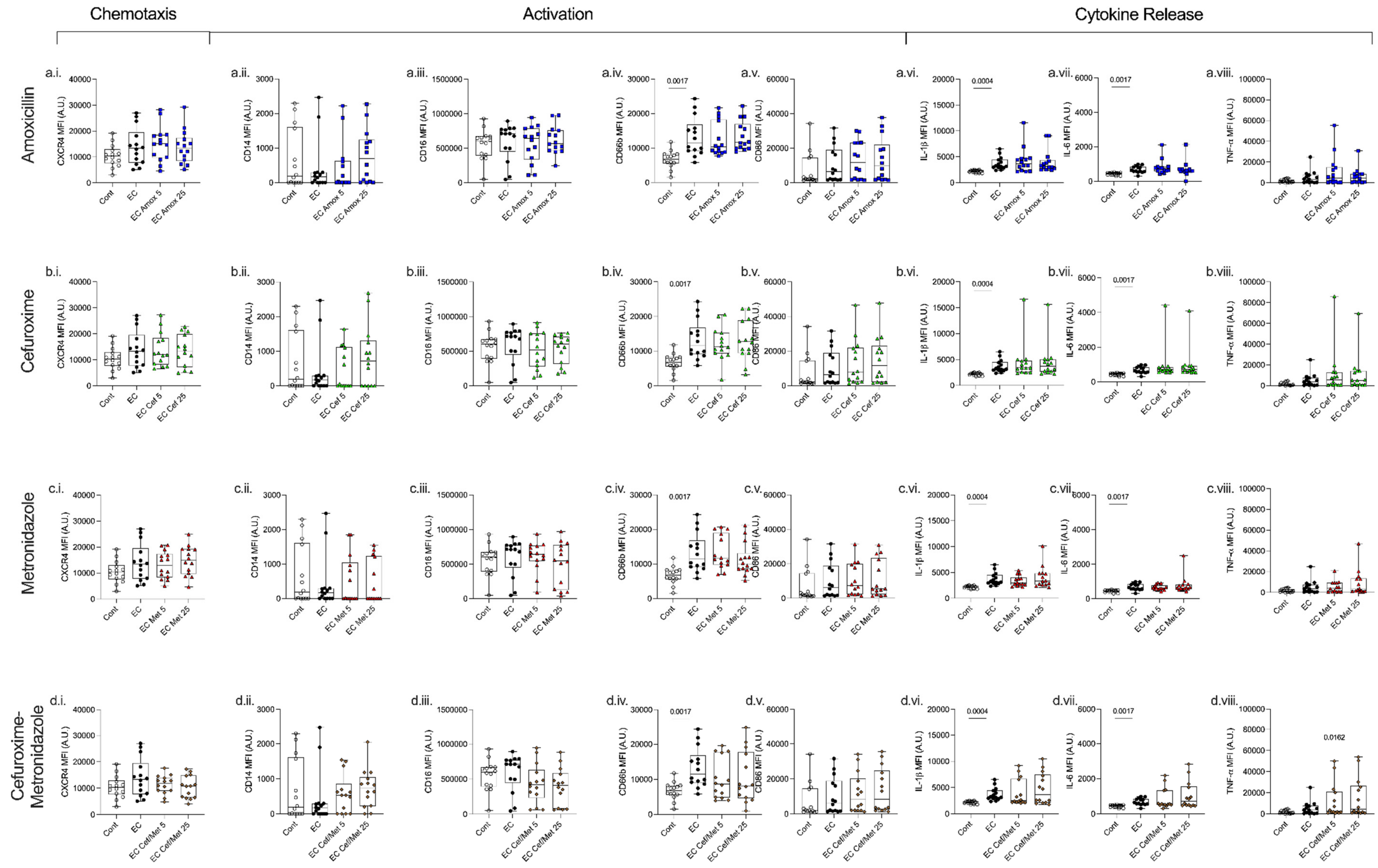

Supplemental Figure S12: Effect of antibiotics on stimulated volunteer granulocyte function

Healthy volunteer (n=16) diluted whole blood was incubated for 6 hours with heat-killed E *coli* (EC) and the effect on granulocyte immune cell markers of amoxicillin (a.), cefuroxime (b.), metronidazole (c.) and cefuroxime-metronidazole (d.) at a concentration of 5 or 25ug/ml was delineated. Immune markers measured include chemokine receptor expression (CXCR4, column i,) cell activation (CD14, column ii., CD16 column iii., and CD66b, column iv.), antigen presentation (CD86 column v.), and cytokine concentration (IL-1β column vi., IL-6 column vii., and TNF-α column viii). Data expressed as median fluorescence intensity measured in arbitrary units (MFI (A.U.)). Individual points represent individual volunteers, horizontal line the median, box the interquartile range and whisker the range. Data compared using Wilcoxon test (control (Cont) vs. EC) or Friedman test without post hoc correction (EC vs. antibiotics). Only $p<0.05$ shown.

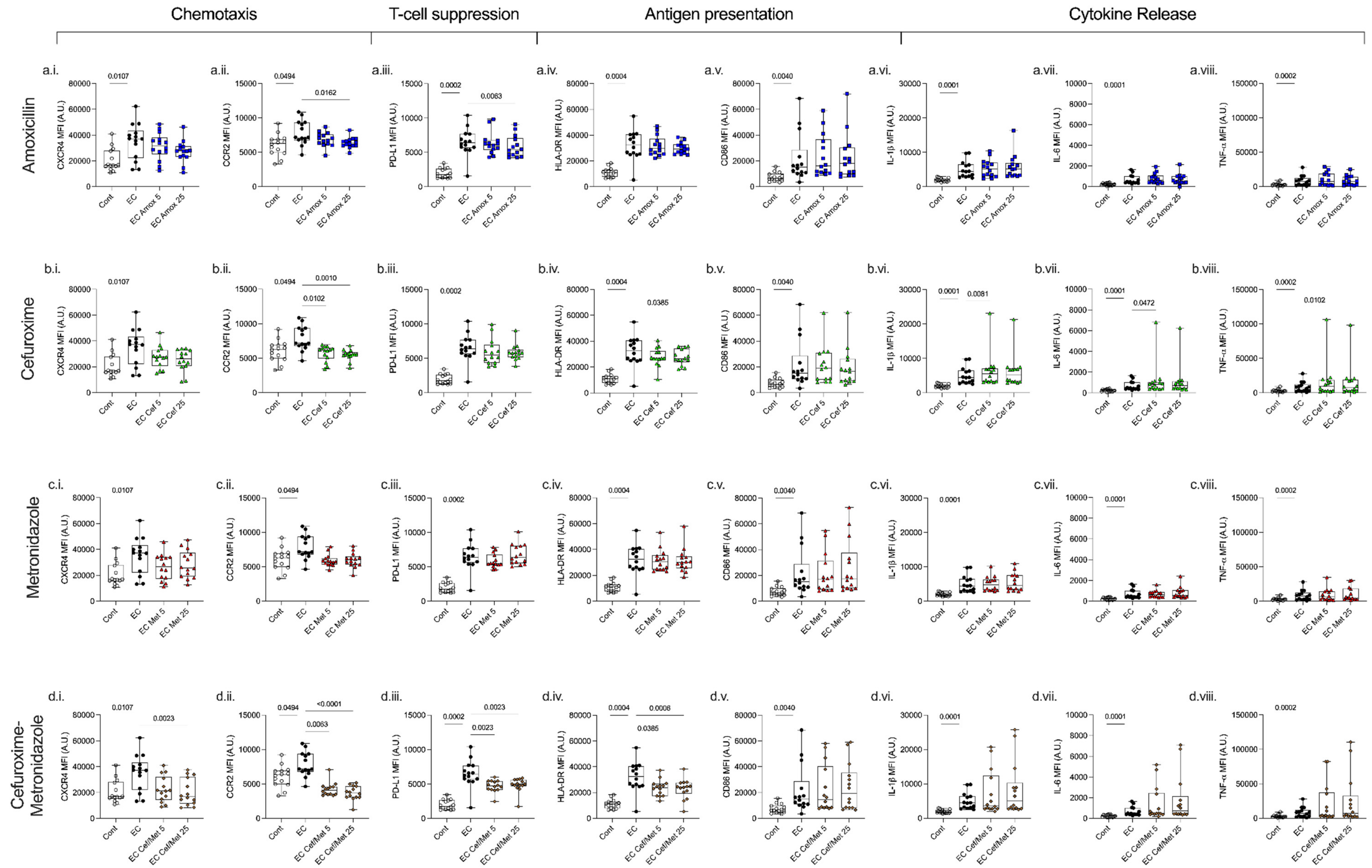

Supplemental Figure S13: Effect of antibiotics on stimulated volunteer monocyte function

Healthy volunteer (n=16) diluted whole blood was incubated for 6 hours with heat-killed E coli (EC) and the effect on monocyte immune cell markers of amoxicillin (a.), cefuroxime (b.), metronidazole (c.) and cefuroxime-metronidazole (d.) at a concentration of 5 or 25ug/ml was delineated. Immune markers measured include chemokine receptor expression (CXCR4, column i, and CCR2, column ii.), T-cell suppression (PD-L1, column iii.), antigen presentation (HLA-DR, column iv. and CD86, column v.), and cytokine concentration (IL-1β column vi., IL-6 column vii., and TNF-α column viii). Data expressed as median fluorescence intensity measured in arbitrary units (MFI (A.U.)). Individual points represent individual volunteers, horizontal line the median, box the interquartile range and whisker the range. Data compared using Wilcoxon test (control (Cont) vs. EC) or Friedman test without post hoc correction (EC vs. antibiotics). Only $p<0.05$ shown.

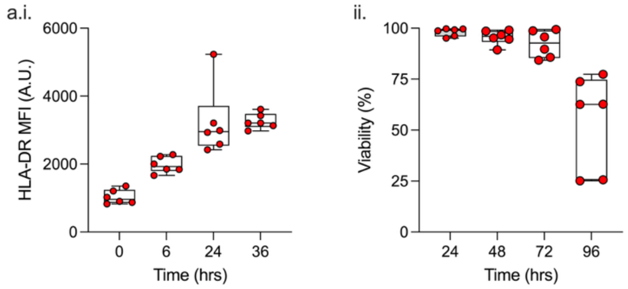

Supplemental Figure S14: Time-course of effect of stimulus on monocyte HLA-DR expression and CD4⁺ lymphocyte viability

Healthy volunteer PBMCs (n=6) were incubated with either (a.i.) heat-killed E *coli* and the effect on classical monocyte HLA-DR expression assessed at 0, 6, 24 and 36 hours, or (a.ii.) CD3/28 beads and the effect on $CD4^+$ lymphocyte viability assessed at 24, 48, 72 and 96 hours. Data expressed as median fluorescence intensity measured in arbitrary units (MFI (A.U.)) or percentage (%) of population. Individual points represent individual volunteers, horizontal line the median, box the interquartile range and whisker the range.
